# Calming Troubled Waters: A Narrative Review of Challenges and Potential Solutions in the Residency Interview Offer Process

**DOI:** 10.5811/westjem.2020.11.49709

**Published:** 2020-12-14

**Authors:** Laura R. Hopson, Mary A. Edens, Margaret Goodrich, Michael Kiemeney, Elizabeth B. Werley, Adam Kellogg, Douglas Franzen

**Affiliations:** * University of Michigan Medical School, Department of Emergency Medicine, Ann Arbor, Michigan; † Louisiana State University Health Shreveport, Department of Emergency Medicine, Shreveport, Louisiana; ‡ University of Massachusetts Medical School – Baystate Health, Department of Emergency Medicine, Springfield, Massachusetts; § Loma Linda University Medical Center, Department of Emergency Medicine, Loma Linda, California; ¶ Penn State Health, Milton S. Hershey Medical Center, Department of Emergency Medicine, Hershey, Pennsylvania; || University of Washington, Department of Emergency Medicine, Seattle, Washington

## Abstract

The rising numbers of residency applications along with fears of a constrained graduate medical education environment have created pressures on residency applicants. Anecdotal evidence suggests substantial challenges with the process of offering residency interviews. This narrative review is designed to identify and propose solutions for the current problems in the process of offering residency interviews. We used PubMed and web browser searches to identify relevant studies and reports. Materials were assessed for relevance to the current process of distributing residency interviews. There is limited relevant literature and the quality is poor overall. We were able to identify several key problem areas including uncertain timing of interview offers; disruption caused by the timing of interview offers; imbalance of interview offers and available positions; and a lack of clarity around waitlist and rejection status. In addition, the couples match and need for coordination of interviews creates a special case. Many of the problems related to residency interview offers are amenable to program-level interventions, which may serve as best practices for residency programs, focusing on clear communication of processes as well as attention to factors such as offer-timing and numbers. We provide potential strategies for programs as well as a call for additional research to better understand the problem and solutions.

## INTRODUCTION

It is increasingly apparent that the process by which interview offers are distributed to medical students in the residency application process is becoming a source of substantial stress to participants and a disruption to their educational environment and personal life. Little direct data exists on the extent of the problem and its impact; however, extensive anecdotal evidence exists and clearly resonates with match participants. These anecdotes from both traditional peer-reviewed and online sources cite problems such as avoiding or compromising participation in educational activities, compromised personal life, and involving multiple friends and family members to manage responses.^1^–^4^ While evidence is mixed as to the predictive value of the residency interview for future performance, it is heavily weighted in candidate decisions, and serves as a de facto gatekeeper for inclusion on a program’s rank order list and potential placement at that residency program.^5^,^6^

Over the past decade and particularly during the past five years, there has been a steady upward trend in the number of residency applications per medical student applicant. US allopathic medical school graduates across all specialties selected have increased from 49 applications in 2014 to 60 in 2018.^7^ Osteopathic graduates in a similar time frame have jumped from 38 to 62 applications.^7^ Residency programs have concurrently experienced a similar rise in application numbers during the same time period, increasing from just over 900 per program to over 1000 in 2016 before levelling off in subsequent years.^8^

Preliminary Electronic Residency Application Service (ERAS) data from the 2019 application cycle continues to show upward trends in application numbers.^9^ This increase in application numbers has not been reflected in a change in match rates.^10^,^11^ There also has not been a significant change in the number of programs on the rank order list (ROL) list required to successfully match among matched applicants.^11^–^13^ As ROL positions are a close surrogate for interviews completed this would argue that the number of interviews needed to successfully match has not increased in proportion to the increased numbers of applications.^14^ However, the perception of an increasingly competitive residency match environment and a desire to avoid the catastrophic “unmatched” state is likely driving application behaviors.^15^

Several proposals to structurally modify the match and application process including limits on numbers of interviews are currently being offered; however, large-scale reform will take time.^16^–^19^ These proposals include strategies such as a multistage match process and preference signaling systems but have not progressed beyond the concept stage. There are potentially factors related to residency interview scheduling practices that reside within the residency program’s control and could be modified to decrease the anxiety and potential disruption to applicants’ lives and educational undertakings.

### Objectives

It was our objective with this narrative review to identify the current problems in the process of offering residency interviews, summarize the available literature on the subject, and provide potential solutions from the literature and expert opinion of the authors. Our goal is to provide residency programs with potential strategies to minimize the disruptive nature of this essential process.

## METHODS

The author group consisted of emergency medicine (EM) faculty with extensive collective experiences as leaders in undergraduate and graduate medical education (UME-GME). The authors were drawn from members and leadership of the Application Process Improvement Committee convened by the Council of Residency Directors in Emergency Medicine (CORD) and have all been involved in specialty-based initiatives to address the recent increase in residency application numbers. We identified articles using a search of PubMed using search strategies described in Table 1. Keywords and Medical Subject Headings (MeSH) terms were reviewed in identified articles to inform the parameters of the search and verify appropriateness of our terms. Results included perspective pieces (majority), observational studies, survey studies, and literature reviews.

We reviewed articles iteratively and then decided by consensus which were relevant to include; any disagreements were adjudicated through the lead authors (LH and DF). General guidelines for inclusion were a focus on the actual interview offer or scheduling process and not on the structure of the day itself, the applicant selection process, or interview outcomes. While our focus was on EM, we screened literature from outside of EM and included articles if they dealt broadly with issues rather than specialty-specific considerations (eg, timing of interviews within a specialty). In addition, the articles had to deal with the United States’ residency match environment. Given the dynamic nature of the process and emerging concerns, we limited our searches to materials within the past 10 years (approximately 2010–2020) to make them relevant to the current day processes. The reference lists of identified articles were also reviewed for potential missed publications with three additional relevant publications (all commentaries and perspective pieces) identified. Due to a paucity of relevant literature within PubMed, we also used similar search terms for web searches using the Google search engine for relevant information and returned multiple blog and forum postings relevant to the topic. These were used to further inform this review. We collectively reviewed the identified literature and developed broad thematic categories through iterative discussion to achieve consensus. No institutional review board review was required due to the absence of human subject involvement.

## RESULTS

There is limited literature evaluating the extent of the problem regarding the current practice of residency interview offers. Overall, the quality of the literature is low, consisting of individual perspectives with occasional single-site or single-specialty studies addressing limited facets of this question. We identified several areas of challenge within the process. These themes include the following: 1) uncertain time from application submission to release of interview offers; 2) disruption caused by release of interview offers; 3) overextending interview offers more than interview slots available; 4) lack of clarity with regard to waitlist/rejection status; 5) interview scheduling mechanisms; and 6) couples match coordination.

## DISCUSSION

Through the literature identified, we classified the issues representing the areas of challenge in the interview scheduling process. We discuss these areas below and attempt to identify potential program-level interventions that may help ameliorate the pressures felt by applicants.

### Problem 1: Uncertain Time from Application to Interview Offers

One of the first decisions programs make that directly affects applicants is the timing of the interview offer. From the program perspective the process is relatively well defined with application review taking up the majority of time between ERAS submission by the applicant and interview offers weeks later. It is easily understood how narrowing the average field of around 900 applications per program down to the initial number of offers can take up this entire period of time.^7^ However, to the applicant who is undergoing this process for the first time, no such insight is available regarding this time-consuming process required to review applications. The timing of when interview offers are released also varies tremendously among specialties ranging from the day ERAS opens to several months later.^20^,^21^ There is even substantial variation within specialties as to when individual programs release interviews. Hence, students are unable to anticipate when to make themselves available for scheduling. While similar data does not exist within EM, a 2016 study of vascular surgery websites showed that fewer than 40% made their interview dates available and fewer than 20% provided any information about their selection processes.^20^ A recent report examining general surgery residencies showed an even worse paucity of information with less than a quarter of websites providing information about interview dates and a mere 3.4% of programs noting interview release dates.^21^

Several potential solutions exist. All of them center around transparency of process and sharing information with the applicant. For example, a consortium of county programs in EM has voluntarily chosen to coordinate a common interview release date.^22^ While such a coordinated initiative may pose substantial logistical challenges to coordinate different program and specialty priorities, there are potentially simple, program-level initiatives. Each program could clearly post its interview offer process on its residency website including date(s) of interview-offer releases and whether the program uses rolling offers with multiple rounds of releases and maintains a waitlist. This information is already being collected unofficially via crowdsourcing on message boards such as Reddit.^23^

In an older study, DeIorio et al found a high prevalence within EM of offering interviews prior to the release of the Medical School Performance Evaluation (MSPE).^24^ It is important to note that this study not only reflects the state in a single specialty but was also performed prior to the MSPE release date being moved to October 1 from November 1. The current extent of this practice is unknown and further confounded by the delayed opening of ERAS caused by the academic disruptions from the COVID-19 pandemic. To level the playing field between applicants, EM programs could collectively agree to only review applications when all data is available, including the MSPE. There may, however, be specific circumstances where early review or interview without a full application are appropriate such as when applicants are known to the program (eg, home medical school or students on a visiting rotation).

### Problem 2: Disruption Caused by Release of Offers

The “first come, first served” model of interview invitations has led to adaptive but dysfunctional applicant behaviors to ensure that they are immediately available to respond when interview offers are released. Students describe finding all available interview spots filled, despite responding to offers within minutes.^4^, ^25^ Such reports create immense anxiety among applicants, to the point of potentially disrupting patient care activities.^1^ Students factor the need to be available into their fourth-year schedule, including avoiding rotations with potentially poor internet service, or surgical rotations where operative time impedes them from responding quickly to an offer. Some literature shows students altering their day-to-day routine such that they are readily available to respond to interview invitations. For example, Sinnott and Johnson describe that “we kept our phones perched precariously on the bathroom sink while showering. We would immediately pull over to the side of the road if our phones vibrated in the car.”^4^ Friends and family members are also affected when they are enlisted to help monitor phones and email accounts.^27^ Such behaviors are, unfortunately, understandable responses to the current system of interview offers.

An obstetrics and gynecology residency has proposed a model to provide applicants a clear window to respond to interview offers with a recommendation of a minimum interval of 48 hours.^4^ Within EM, Klein et al described a two-phase strategy of first informing an applicant of their interview offer and the date and time of availability for online scheduling, followed by opening of the online scheduling system at a time designed to minimize interference with personal life and clinical/school obligations. The authors, however, did not provide any outcomes data related to this approach.^22^ Such strategies allow students not only time to respond but also time to consider the offer, potentially limiting “interview hoarding” and students accepting an interview simply because they are unsure whether they will get other offers. However, both strategies require programs to send out only as many invitations as they can accommodate.

Programs may consider these models as interventions to decrease the disruption caused by the current method of interview offers. Clear, advance information for applicants regarding when interviews offers will be released, whether this applies just to initial offers, or whether a program uses a staged release of offers could be provided. Students could thus ensure they were available for those dates and times. Initiatives on a specialty-level may also promote and publicize universal release dates. Early notification of a definitive rejection may also allow students closure and decrease the number of programs a student needs to monitor.

### Problem 3: Imbalance of Interview Offers and Available Positions

The practice of extending more interview offers than actual positions available for interviews creates an increased sense of panic for students that they will be shut out of an interview.^19^,^20^,^21^ National Residency Matching Program (NRMP) applicant surveys demonstrate a multispecialty, five-year trend of dramatically increasing application numbers, slowly increasing numbers of interviews attended, and minimally increasing numbers of programs ranked.^29^–^31^ From this, we can extrapolate that applicants must be declining significantly more interview offers than previously. There is a dearth of data from programs regarding their ratio of interview offers vs actual interview positions available with publications within subspecialties of surgery providing the only hard data available.^25^,^26^ Anecdotal data is extensive across specialties and suggests that the problem of more interview offers than slots is extensive.^3^,^4^,^27^

Solutions will require new approaches to the interview offer process on the program level. On the offer date, programs may choose to intentionally release no more offers than actual interview slots with the intention of filling the remaining openings later. Programs may also consider a purposefully staged release of offers, to allow a balance between applicant decisions and the program’s desire to have a full interview schedule. The program of one of the authors has several years’ experience with this approach and reports no difficulty filling interview positions with high-quality candidates (oral communication, Laura R. Hopson, MD, January 2020). As noted earlier, regardless of the strategy of interview offers pursued, clear communication about expectations regarding how long the candidate may take to accept or decline the offer is beneficial to the applicants. Finally, programs may want to consider allowing candidates to place themselves on the waitlist for interview dates that may be currently full.

### Problem 4: Lack of Clarity Around Waitlist Status or Rejection

Applicants have significant stress related to when interview offers are extended. There is a distinct challenge for applicants who are waitlisted or rejected. There is little literature dedicated to this topic; however, online student forums contain extensive concerns and frustrations related to these situations, including uncertainty of their status, unknown likelihood of getting an interview from the waitlist, and uncertainty stemming from little to no communication regarding an applicant’s waitlist or rejection status.^19^,^32^–^36^

As with interview offers, residency programs may consider an openly published date for notifying applicants of their position on the waitlist. This communication may include an estimate of the likelihood of receiving an interview offer from the program as well as a time frame in which interview offers may be granted. Sample texts for these communications are included in the Appendix. Similarly, clear communication to applicants who will not receive an interview offer may also be of benefit. Literature from general surgery suggests that very few programs provide this valuable information to applicants.^21^ Ideally, the program could also publish a date for notification of rejection status.

### Problem 5: Mechanism of Scheduling

There is substantive work supporting the use of online scheduling programs for residency interviews. Studies by Wills et al and Hern et al in 2015 and 2016 within EM and similar work in surgery by Hoops et al in 2018 showed markedly increased applicant satisfaction and decreased time spent with use of online interview scheduling systems as opposed to direct communication.^37^–^39^

The exact system is likely unimportant but considerations of functionality, ease of access, and dissemination within the specialty to allow for easy scheduling coordination for applicants should be entertained. In the interests of clarity, the system used should be clearly identified in advance, thus allowing applicants to adjust email spam filters and thereby avoid missed communications.

### Problem 6: Couples Match Coordination

The couples match presents a unique set of challenges around interview offers. Coordinating dual careers increases the baseline emotional and financial stress of this process.^40^–^42^ NRMP data, however, indicates that couples had a better than 90% success rate of matching each year since 1984, when the couples match first became an option. Additionally, for US seniors, who comprise approximately 69% of couples within the Match, match rates are similar to their non-couple classmates.^10^

However, there are some who question whether this data may be skewed in a practical sense. It is not a large jump to assume that the two members of a couple want to be in proximity to each other. One commentary, describing a married couple who matched at programs far from each other, questions the success the NRMP claims regarding the couples match: “Since coupled applicants are allowed to submit rank lists with limitless match combinations, including those that would place them hundreds of miles apart, this ‘success’ rate fails to account for whether a couple matched within the same geographical area.”^43^ Any number of factors may impact an applicant participating in the couples match, including desired geographic location, available programs for both applicants within a given location, type of program desired, competitiveness of the partner’s desired specialty, association with a preliminary year, and strength of each applicant’s application.

Advice from applicants who successfully survived the couples match includes having a strategy based on the priorities of both members individually and as a couple.^43^ Alvin and Alvin also suggest enhanced intra- and inter-institutional communication between programs to which couples are applying regionally, as well as the possibility of a “trigger” notification to the other program if one half of the couple is offered or accepts an interview at the same institution or potentially within a specified region.^43^ Such mechanisms would have to balance considerations of privacy and convenience.

## LIMITATIONS

We fully acknowledge that our study is limited by the lack of evidence-based data available on this topic and draws heavily on anecdotal experiences. In our search, there appears to be more content from the applicant-side than the program-side of the process. This may cause the magnitude of problems to be overemphasized. Further review is likely warranted with a focus on the program perspective in order to balance these initial findings. Our author group represents diverse stakeholders across the UME-GME spectrum but does come from a single specialty, and additional insights may exist in other realms. Finally, while this piece grew out of work done by the Application Process Improvement Committee convened by CORD, the conclusions contained represent the views of the authors and are not intended as a formal policy statement by the organization.

## CONCLUSION

Substantial anecdotal evidence exists that the process of offering residency interviews has become disruptive on many levels. Little formal literature exists to categorize the magnitude of the problem. We propose relatively simple interventions at the program level. Many of these echo suggestions starting to be made within other medical specialties.^21^,^26^ These suggested interventions may help to alleviate challenges for applicants without creating undue burdens on training programs. In addition, with the ongoing uncertainty and disruption in medical education caused by the coronavirus pandemic and the potential to impact the residency application environment, having programs adopt best practices, particularly those focused around clear communication and expectations, can only benefit the process. We strongly support additional research to define and quantitate the impact of residency interview offer practices and potential interventions.

## Supplementary Information



## Figures and Tables

**Table 1:** Search strategy and results regarding the current system of offering interviews to residency applicants

Terms used	Results returned	Results within 2010–2020 timeframe	Relevant results*
Internship and residency and Interview as topic and Offers	16	13	1
Graduate Medical Education and Interviews as topic and Offers	8	7	0
Graduate Medical Education and Interviews as topic	318	238	0
Residency Interview Scheduling	638	330	8
Graduate Medical Education and Interviews as topic and appointment and schedules	9	7	3

* Some publications may be identified in more than one search.
