# Supplementary material for: Calming Troubled Waters: A Narrative Review of Challenges and Potential Solutions in the Residency Interview Offer Process
Source: West J Emerg Med. 2020 Dec 14;22(1):1–6. doi: 10.5811/westjem.2020.11.49709 (PMC7806321; doi:10.5811/westjem.2020.11.49709)
Supplement: Supplementary file 1 [file wjem-22-1-s001.docx]

**Appendix:** Examples of Recommended Communications to Applicants Regarding Status and Expectations

**Interview Offer**

Thank you for applying to our Residency Program. We have reviewed your ERAS application and are excited to offer you a position to interview at our program!

We will open scheduling for interviews on Monday, October XX at 8pm Eastern Time through an on-line scheduling program. You will receive a second email invitation at that time to access the system. This invitation will be active for 72-hours after its release. We do not offer more interviews than we have positions available. We will guarantee you have an interview position, provided we receive your response within the 72-hour window. Please take your time considering this offer - we want to ensure that interviewees are genuinely interested in coming to our program.

The times and dates of our program interviews are available on our website at Link

We realize this process is stressful and out of respect for all applicants, we ask the following:

- Please decline the invitation or notify us directly if you do not wish to interview at our program. This will allow us to rapidly offer a position to another interested applicant
- Please provide a minimum of a 14-day notice if you decide to cancel your interview once scheduled. This will allow time for another interested applicant to be scheduled.

We will be sending you additional information once your interview is scheduled. In the meantime, please feel free to contact us if we can provide additional assistance.

**Waitlist**

Thank you for applying to our Residency Program. We have reviewed your ERAS application and would like to offer you a position on the waitlist for an interview. We view you as a strong candidate for the residency and would be excited to have you interview as soon as a space is available. Approximately, 50% of our wait-listed applicants will be offered an interview position

If you do not want to remain on the waitlist, please let us know via email at residency@med.edu.

We will notify all waitlist candidates of their final status by the first week of December.

**Rejection**

Thank you for applying to our Residency Program. We have reviewed your ERAS application and regret to inform you that we are unable to offer you an interview. We wish you the best.
